# High-Temperature Mechanical Properties of Stress-Relieved AlSi10Mg Produced via Laser Powder Bed Fusion Additive Manufacturing

**DOI:** 10.3390/ma15207386

**Published:** 2022-10-21

**Authors:** Dirk Lehmhus, Thomas Rahn, Adrian Struss, Phillip Gromzig, Tim Wischeropp, Holger Becker

**Affiliations:** 1 Fraunhofer Institute for Manufacturing Technology and Advanced Materials IFAM, Wiener Straße 12, 28357 Bremen, Germany; 2 Fraunhofer Institute for Additive Production Technologies IAPT, Am Schleusengraben 14, 21029 Hamburg, Germany; 3 BDG-Service GmbH, Hansaallee 203, 40549 Düsseldorf, Germany

**Keywords:** additive manufacturing, Laser Powder Bed Fusion (LPBF), Laser Beam Melting (LBM), aluminum alloy, casting, high-pressure die casting (HPDC), compound casting, mechanical properties, high-temperature properties

## Abstract

The present study is dedicated to the evaluation of the mechanical properties of an additively manufactured (AM) aluminum alloy and their dependence on temperature and build orientation. Tensile test samples were produced from a standard AlSi10Mg alloy by means of the Laser Powder Bed Fusion (LPBF) or Laser Beam Melting (LBM) process at polar angles of 0°, 45° and 90°. Prior to testing, samples were stress-relieved on the build platform for 2 h at 350 °C. Tensile tests were performed at four temperature levels (room temperature (RT), 125, 250 and 450 °C). Results are compared to previously published data on AM materials with and without comparable heat treatment. To foster a deeper understanding of the obtained results, fracture surfaces were analyzed, and metallographic sections were prepared for microstructural evaluation and for additional hardness measurements. The study confirms the expected significant reduction of strength at elevated temperatures and specifically above 250 °C: Ultimate tensile strength (UTS) was found to be 280.2 MPa at RT, 162.8 MPa at 250 °C and 34.4 MPa at 450 °C for a polar angle of 0°. In parallel, elongation at failure increased from 6.4% via 15.6% to 26.5%. The influence of building orientation is clearly dominated by the temperature effect, with UTS values at RT for polar angles of 0° (vertical), 45° and 90° (horizontal) reaching 280.2, 272.0 and 265.9 MPa, respectively, which corresponds to a 5.1% deviation. The comparatively low room temperature strength of roughly 280 MPa is associated with stress relieving and agrees well with data from the literature. However, the complete breakdown of the cellular microstructure reported in other studies for treatments at similar or slightly lower temperatures is not fully confirmed by the metallographic investigations. The data provide a basis for the prediction of AM component response under the thermal and mechanical loads associated with high-pressure die casting (HPDC) and thus facilitate optimizing HPDC-based compound casting processes involving AM inserts.

## 1. Introduction

Additive manufacturing (AM) processes of the Laser Powder Bed Fusion (LPBF) type, which is sometimes also referred to as Laser Beam Melting (LBM), tend to rely on the same choice of alloys as other production technologies that are based on the melting of metals. When it comes to aluminum alloys, a standard choice in this respect are compositions close to certain eutectics—not the least the Al-Si one with a melting temperature of roughly 570 °C, to which additions such as copper, magnesium and zinc add strength by facilitating precipitation hardening treatments. Aluminum casting alloys, e.g., of the near-eutectic AlSiMg type, are typically not subjected to service temperatures above 150 °C because of their limited strength under such conditions and the adverse effect on heat treatment states such as T5 or T6. However, in certain production scenarios, these temperature levels may be exceeded by a large margin. One example of this type is compound casting, in the course of which separately manufactured components are joined to and/or integrated in cast components by means of the casting process itself [1,2]. Compound casting processes as such have been used across a wide range of material combinations and casting processes. Among the former, the combination of cast aluminum with dissimilar metals such as magnesium [3], copper [4,5] or steel [6,7] is common, but combinations of aluminum alloys with each other have also been studied in significant detail [8,9,10]. Major difficulties in realizing these combinations arise from the fact that the temperature of the melt during casting typically approaches or even exceeds the solidus temperature of the insert. For this reason, in contrast to aluminum-copper or aluminum-steel, aluminum-aluminum compound casting is most commonly associated with the high-pressure die casting (HPDC) process, as in this case, fast extraction of heat from the melt and resulting high solidification rates prevent excessive melting of the insert.

The motivation for using AM components as inserts in compound casting rests upon the tremendous geometrical flexibility of this manufacturing process. The ensuing geometrical freedom can be employed to realize complex regions of the final component without the need for extra complexity on the part of the die, provide optimized internal reinforcements [7] or improve the bonding between both partners by enabling complex micro- to macro-scale form fit solutions. Nowadays, combinations of aluminum-based, additively manufactured hollow structures and the HPDC process move into the focus in an E-mobility context: Aluminum is the material of choice for realizing cooling channels in electric power train components such as battery, power electronics or electrical engine housings. In large-scale series production of such components, HPDC is the preferred production process for economic reasons. Direct realization of internal cavities, however, fails due to a lack of lost cores that can withstand the harsh processing conditions. The alternative is the integration of tubes, but once again the combination of pressure (typically 600–1200 bar) and temperature (typically above 700 °C, depending on the alloy) means that only aluminum tubes stabilized by fillers such as salt, which need to be laboriously removed after casting, will withstand the load [11,12]. AM, in contrast, facilitates solutions in which internal structures within such cooling channels can serve a double purpose by being designed, on the one hand, to increase resistance against the thermomechanical loads associated with the casting process, and on the other to optimize heat transfer between casting and cooling media. If successful, this would allow eliminating the filler and its removal from the process chain while at the same time improving the cooling performance. Initial experiments in this respect already hint at the high potential of this approach [13].

**Figure 1:**
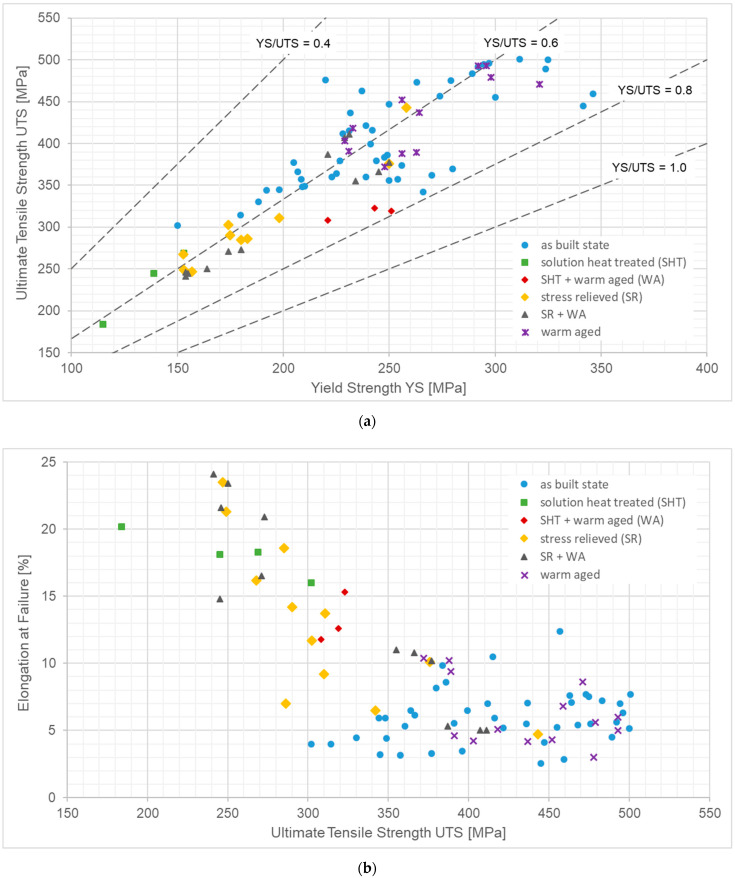
Overview of room temperature properties of AlSi10Mg produced via the LPBF process as reported in the scientific literature [16,18,21,22,26,27,28,29,30,31,32,33,34,35,36,37,38,39]: (**a**) ultimate tensile strength (UTS) plotted vs. yield strength (YS), and (**b**) elongation at failure plotted vs. UTS. The data cover different printing directions, process parameters and heat treatment states, namely as-built (AB), stress-relieved (SR), solution heat-treated (SHT) and warm-aged (WA), plus combinations of these. The individual values are presented in Table A1 in Appendix A together with a brief explanation of the respective boundary conditions.

The prerequisite for realizing such concepts is detailed knowledge of the elevated temperature properties of the respective AM structures. To extend this knowledge is the primary aim of the present study. AlSi10Mg is a perfect candidate alloy for evaluating some of the above assumptions, as it is a standard material both in HPDC and LPBF. This suggests availability of a broad property database for both processes. Furthermore, when used in a compound casting context, problems which may arise from dissimilar metal joints are eliminated. AlSi10Mg powders are offered by several suppliers, including the AM equipment manufacturers themselves, for processing according to the LPBF method. The characteristics of this material have thus been studied previously and in detail by several authors. Among others, multiple studies are available which investigate the influence of factors such as scanning parameters [14,15], build orientation [14,16,17,18], post-processing including heat treatment [19,20,21,22] or state of powder, e.g., after recycling [23,24,25], on room temperature mechanical properties. Figure 1 provides an overview of the findings based on yield strength, ultimate tensile strength and elongation at failure and illustrates the effect of heat treatments on room temperature properties. The underlying individual values are presented in Table A1 in Appendix A together with additional information on relevant processing conditions, heat treatment states, etc., as well as links to the corresponding data sources. 

As expected, despite the spread of parameters and hence material conditions, the data show a rough dependency between yield and ultimate tensile strength, with the ratio between both approaching 0.8 as the latter increases (see Figure 1a). Similarly, and as is generally known for metallic materials, as long as the same fundamental mechanisms of plastic deformation apply, a higher UTS implies lower ductility, as expressed here by a reduction in elongation at failure (see Figure 1b). From an application perspective, the diagrams illustrate the considerable area within which material properties can be adjusted by an appropriate choice of primary manufacturing and post-processing parameters and boundary conditions.

There are, in general, only very few studies focusing on the high-temperature characteristics of additively manufactured aluminum alloys. An exception to this rule is a recent study by Uzan et al., against which the present findings will be benchmarked [40]. Further reference data can be obtained from Cao et al., who also provide a thorough study on microstructural evolution at elevated temperatures up to 400 °C [41], while Liu et al. tested LPBF samples at room temperature and 200 °C [39]. Additional information on microstructure evolution up to 180 °C has been collected by Fousová et al. [42]. In a compound casting context, freedom from residual stresses is an important aspect, as these could lead to distortion of any integrated structures during casting. Hence, the present study focuses on material conditions promising low levels of residual stress. For this reason, samples were produced at elevated build platform temperatures of 200 °C and furthermore stress-relieved for 2 h at 350 °C following production. None of the aforementioned studies deal with the high-temperature characteristics of such material conditions. These are therefore covered in the present study, which provides quantitative data on tensile properties as well as observations on failure mechanisms for different build orientations (0°, 45° and 90°) and test temperatures (room temperature (RT), 125, 250 and 450 °C), thus extending the state-of-the-art in this direction.

## 2. Materials and Methods

Table 1 provides an overview of the composition of the gas-atomized AlSi10Mg powder used in this study, which was supplied by SLM Solutions Group AG, Lübeck, Germany, listing specification information as well as measured data issued by the supplier [43]. The reader should note that the specified composition refers to the virgin powder in its as-delivered state. The authors’ determination of the elemental composition relied on optical emission spectroscopy (OES) using a Thermo Scientific (Waltham, MA, USA) ARL 3460 Metals Analyzer system and was performed not on the powder, but on the printed samples. Results are summarized in Table 1 below. For the present experiments, powders were employed which had already been recycled several times. The influence of this procedure may show in an increased oxygen content. When setting up the experimental plan, it was assumed that the embedded oxides might help improve the high-temperature characteristics, as the respective performance of ceramic particle-reinforced Al-based metal matrix composites (MMCs) suggests. This notion is partially supported by Weiss et al., who showed that improvement in room temperature yield and ultimate tensile strength can indeed be observed in specimens based on recycled powders [24]. To verify this assumption, oxygen content was determined on printed samples using a trace element analyzer of type ONH2000 supplied by Eltra GmbH, Haan, Germany. Average oxygen content was found to be 0.0402 wt.% at a standard deviation of 0.001 wt.%.

The powder particles are of spherical shape, and the powder is specified as particle size grade 20–63 µm. Actual determination of particle size revealed a mean diameter of 47.70 µm, with d_10_, d_50_ and d_90_ values of 25.76, 44.26 and 74.85 µm, respectively, according to supplier measurements performed on the actual batch used in the present study.

Tensile test samples were produced by means of Laser Powder Bed Fusion (LPBF) using an EOS M290 system originating from EOS GmbH, Krailing, Germany. The system is equipped with an F-Theta lens with a focus diameter of 100 µm. Sample production was performed without interruption of the build process. For powder application, a silicone coater lip of type SLM 250 as produced by SLM Solutions Group AG, Lübeck, Germany, was employed at a speed of 100 mm/s. This was made possible via an adapter designed and manufactured in-house by Fraunhofer IAPT. Information on processing parameters is summarized in Table 2. As general measures, the scanning direction was rotated by 67° from layer to layer, skywriting was activated, and a stripe exposure strategy was used. The temperature of the build platform was kept at 200 °C. The parameter selection was aimed at achieving a compromise between the build rate and part quality, e.g., expressed in density and not at reaching the highest density at all costs, as this would only be possible at reduced productivity. The choice of speed rather than quality-centered parameters reflects the requirements of the envisaged application, HPDC-based compound casting, and is aimed at leveling the economic imbalance between the two manufacturing processes. 

**Figure 2:**
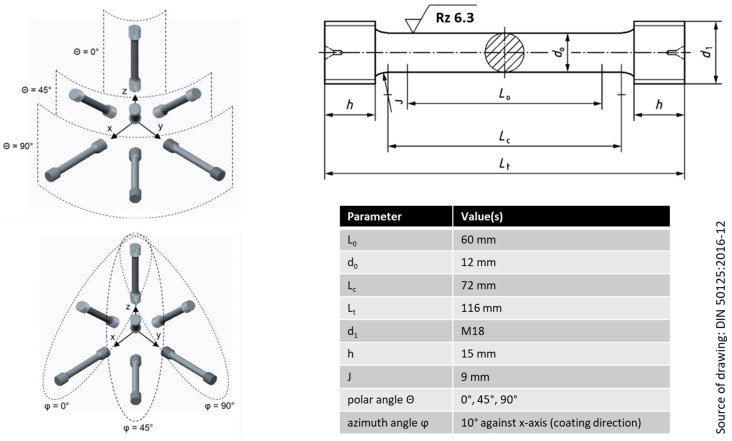
Samples’ dimensions according to DIN 50125:2016-12 and explanation of the build orientation [44]. Note that in the sketches to the left, the *z*-axis is the vertical direction, while the *x*-axis is the re-coating direction in the present experiment and the plane of the build platform is spanned by the *x*- and *y*-axes together. Θ is the polar and φ is the azimuth angle.

Primary process parameters were not varied between samples, however, identical numbers of samples were produced at three different polar angles, namely 0°, 45° and 90°, while the azimuth angle was kept constant at 10°. The respective orientations are presented in Figure 2 together with the geometry and dimensions of the samples produced, which match the DIN 50125:2016-12 specification [44].

All samples were tested in the condition as-manufactured, i.e., no machining or polishing of surfaces was applied, with the exception of three additional samples turned from cylinders of slightly larger dimensions manufactured at a polar angle of 0°. These were tested at room temperature to estimate the influence of surface characteristics on the tensile test results by comparing the strength data obtained to the corresponding samples tested in as manufactured state. 

As described in the Introduction Section, the application scenario motivating the experiments was integration of AM components in HPDC parts for realization of cooling channels, as described by Lehmhus et al. [13]. In the respective HPDC casting campaigns, the aim was to study deformation and compression in relation to casting conditions. To eliminate an influence of residual stresses originating from the AM process, it was decided to stress-relieve the respective components, and hence also the tensile test samples, the investigation of which is discussed here. Stress relieving took place in a batch furnace with samples still on the build platform. The dwell time was 2 h at 350 °C, preceded by a slow temperature ramp-up lasting one hour. At the end of the treatment, samples remained in the furnace to cool down to room temperature. 

**Figure 3:**
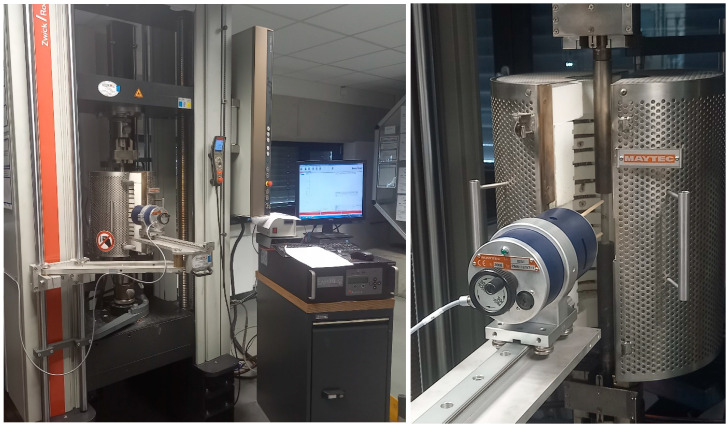
Test setup for high-temperature tensile tests showing sample position and furnace as well as the extensometer used for the experiments. (**Left**) Overview including part of the Zwick Roell Z250 test device, and (**right**) detailed view of the extensometer and sample.

Tensile tests were performed at BDG services GmbH using a Zwick Roell universal testing device of type Z250. Test conditions were in accordance with DIN EN 50125 with respect to room temperature and DIN EN ISO 6892-2 for elevated temperature experiments. Data acquisition and evaluation, i.e., determination of the primary test results such as yield stress, ultimate tensile stress, etc., relied on Zwick Roell’s software testXpert II. Heating of samples was performed via a custom three-zone furnace enclosing the sample during testing originating from Maytec Mess-und Regeltechnik GmbH, Singen, Germany. Strain measurements were performed using a high-temperature extensometer of type PMA-12 supplied by the same company. Figure 3 depicts the tensile test setup as used at all test temperatures, including the strain measurement system.

The time required for heating up of the samples and the duration of immersion at the final temperature prior to the actual test amounted to approximately 20 min for testing at 125, 250 and 450 °C. All tests were performed at a constant strain rate of 0.001 s^−1^. Consequently, depending on the actual duration of the test, samples tested at elevated temperature were subjected to a further heat treatment during testing. The respective times ranged from an average of 88 s for experiments performed at 125 °C, 151 s for 250 °C and 261 s for 450 °C. The increase of the duration with temperature reflects the rise in ductility observed at higher temperatures, as expressed in the observed elongation at failure.

Vickers hardness measurements were performed according to DIN EN ISO 6507-1 on metallographic sections of the respective samples at a force level of 0.981 N [45]. 

Scanning electron microscopy (SEM) investigation of fracture surfaces relied on a Thermo Fisher Phenom XL Desktop system. Metallographic investigations used standard equipment for sample cutting and polishing. Etching of metallographic sections was performed with MIL etchant, a combination of diluted hydrochloric, nitric and hydrofluoric acid at concentrations of 1.5, 2.5 and 1.0 mL per 100 mL of etchant, with the balanced made up by distilled water. For elemental mappings based on energy-dispersive X-ray spectroscopy (EDX), an EDX detector of type X-Max 50 in a Zeiss EVO MA10 SEM (Oberkochen, Germany) was employed at 20 kV and a working distance of 8.5 mm. Vickers microhardness measurements were performed according to DIN EN ISO 6507-1 by means of a Leco Microhardness tester LM248AT at a load of 100 g force or 0.981 N.

## 3. Results

### 3.1. Mechanical Properties

**Figure 4:**
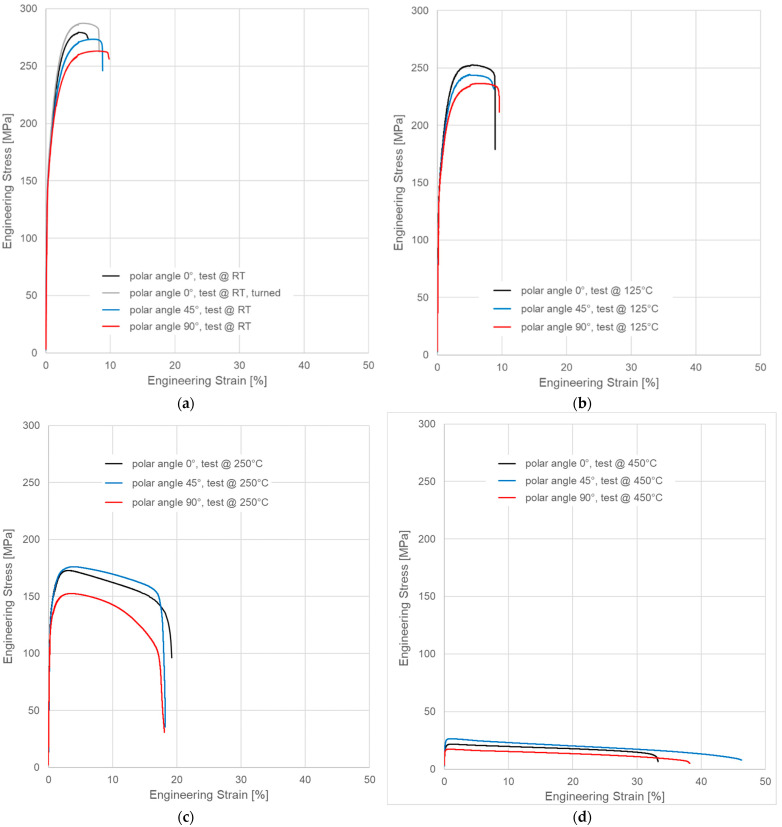
Representative engineering stress vs. strain curves for different build orientations and test temperatures: (**a**) room temperature data, (**b**) test temperature 125 °C, (**c**) test temperature 250 °C and (**d**) test temperature 450 °C. Note that *x*- and *y*-axis scaling are identical in all cases to allow for direct comparison of the diagrams.

Figure 4 depicts exemplary technical (engineering) stress–strain curves measured at room temperature (RT, Figure 4a), 125 (b), 250 (c) and 450 °C (d). Each diagram shows a representative curve for each of the build orientations compared. Figure 4a also includes data for a sample machined from a cylindrical specimen produced at a polar angle of 0° using the same parameters as in the case of all other samples. The same scaling of *x*- and *y*-axes has been adopted for all individual diagrams to make the differences in terms of strength and ductility more evident.

As can also be seen in Figure 4, the influence of surface quality was limited. Table 3 below lists average room temperature values as well as associated standard deviations of yield strength, ultimate tensile strength and elongation at failure measured on as-manufactured and turned samples all printed standing upright, i.e., at a polar angle of 0°. Deviations in terms of strength reach 2.1% for yield strength and 3.1% for ultimate tensile strength, using the values obtained for the turned samples as a reference. Elongation at failure, on the other hand, dropped 24.2% in as-manufactured compared to turned samples.

**Figure 5:**
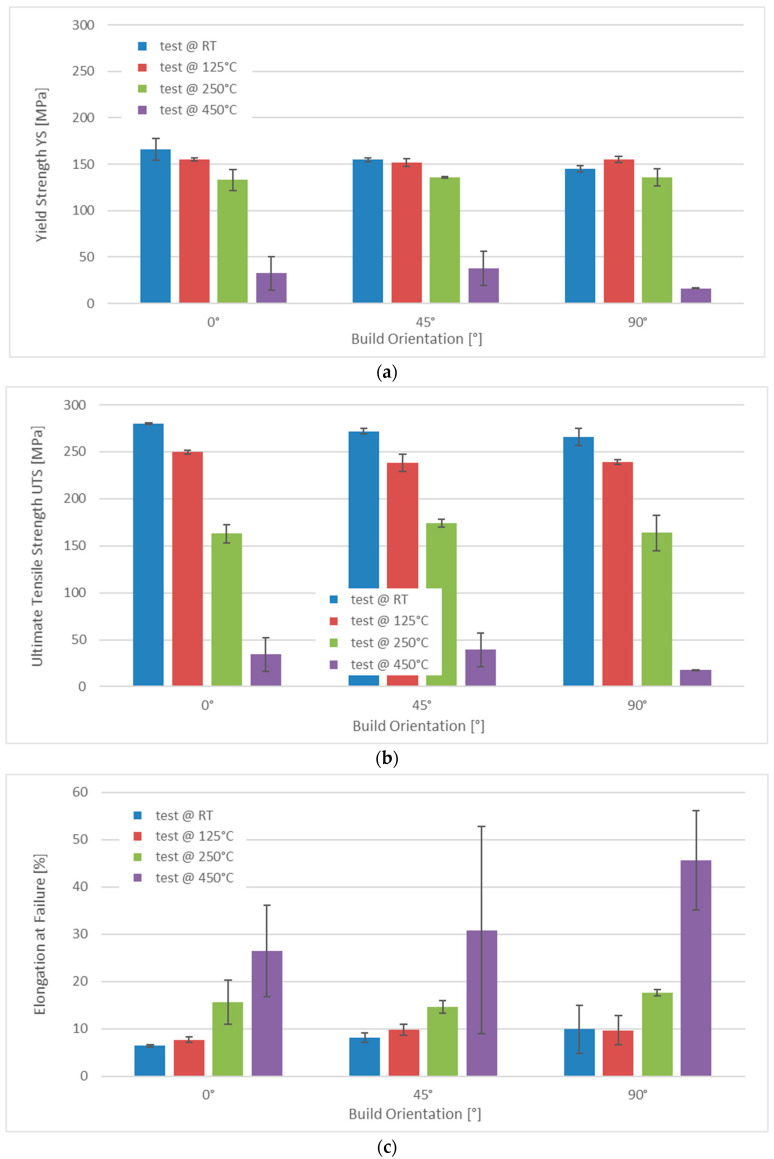
Overview of results of tensile tests performed at room temperature (RT), 125, 250 and 450 °C for three different build orientations, namely polar angles of 0°, 45° and 90°: (**a**) yield strength, (**b**) ultimate tensile strength and (**c**) elongation at failure.

Figure 5 summarizes the results of tensile tests performed at room temperature, 125, 250 and 450 °C on samples with polar angles of 0°, 45° and 90°. The highest strength was observed in samples produced standing upright (0° polar angle)—in terms of ductility, the order was reversed. As expected, strength dropped with the increasing temperature, while ductility as expressed by elongation at failure increased. Up to the level of 250 °C, the reduction in yield strength was limited. In contrast, the relative difference in ultimate tensile strength between samples tested at 125 and 250 °C was significantly larger than in the case of yield strength. The respective values were, in the case of yield strength, 14.2% at 0°, 10.5% at 45° and 12.7% at 90° polar angle compared to 34.8%, 26.9% and 31.7% for ultimate tensile strength. At 450 °C, yield and ultimate tensile strength were reduced to less than 40 MPa in all cases, undercutting the related values obtained at 250 °C in a range from 75.5% (YS, 0°, RT) to 89.3% (UTS, 90°, 450 °C). High-temperature measurements produced large scatter, expressed in high values of the standard deviation. Averaged data for all test conditions as well as the values of the standard deviations are collected in Appendix A, Table A2. 

### 3.2. Microstructure and Fracture Surfaces

The following section contrasts microstructures as well as fracture surfaces observed in relation to the main test and production parameters, namely the test temperature and the build orientation.

**Figure 6:**
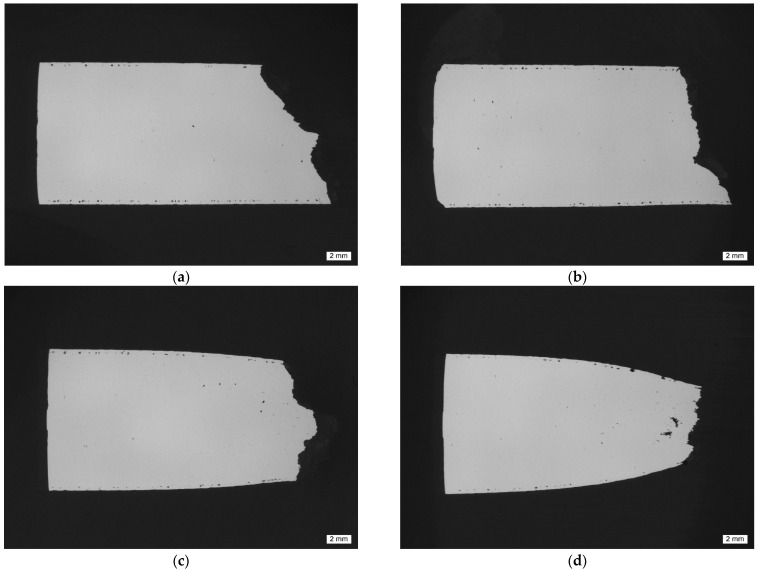
Macroscopic images of unetched metallographic sections of tensile test samples of polar angle 0° tested at increasing temperatures: (**a**) RT, (**b**) 125, (**c**) 250 and (**d**) 450 °C. The images provide an impression of the level of porosity as well as highlighting the necking, which increases with the increasing test temperature from (**a**–**d**). The sample tested at 450 °C also showed evidence of pore growth in the plastic region adjacent to the fracture location.

**Figure 7:**
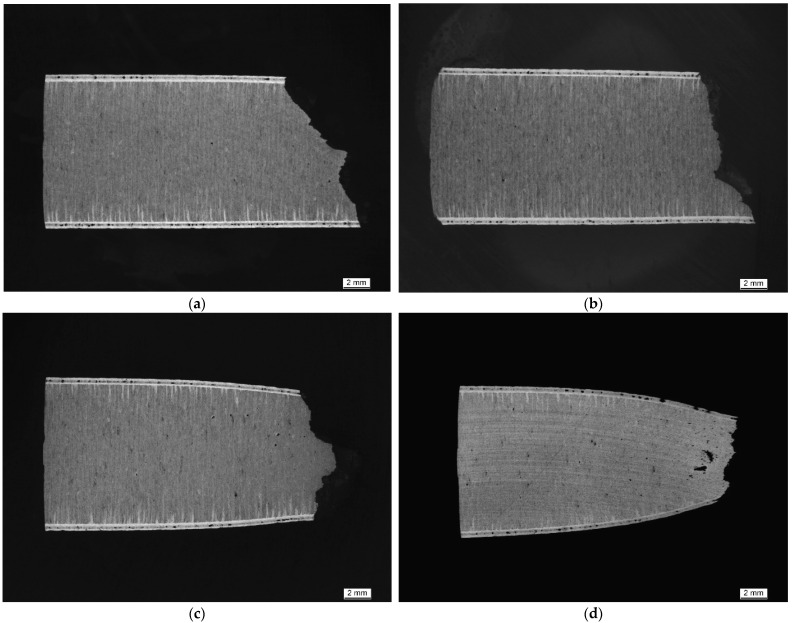
Macroscopic images of metallographic sections of tensile test samples produced at a polar angle of 0° and tested at increasing temperatures, after grain boundary etching using MIL etchant: (**a**) RT, (**b**) 125, (**c**) 250 and (**d**) 450 °C. Figures show increasing levels of necking parallel to the increasing test temperature. Etching highlights the orientation of the individual layers, perpendicular to the direction of force in this case, as well as the boundary layer.

Figure 6 and Figure 7 depict the metallographic cross-section of the fracture region for samples built at a polar angle of 0° and tested at room temperature (RT, a), 125 (b), 250 (c) and 450 °C (d). While Figure 6 contains images of unetched samples, highlighting changes of their outer contour, Figure 7 shows the same samples in the etched condition. The latter images clearly show the layered build-up of the specimens and underline their orientation relative to the loading direction.

**Figure 8:**
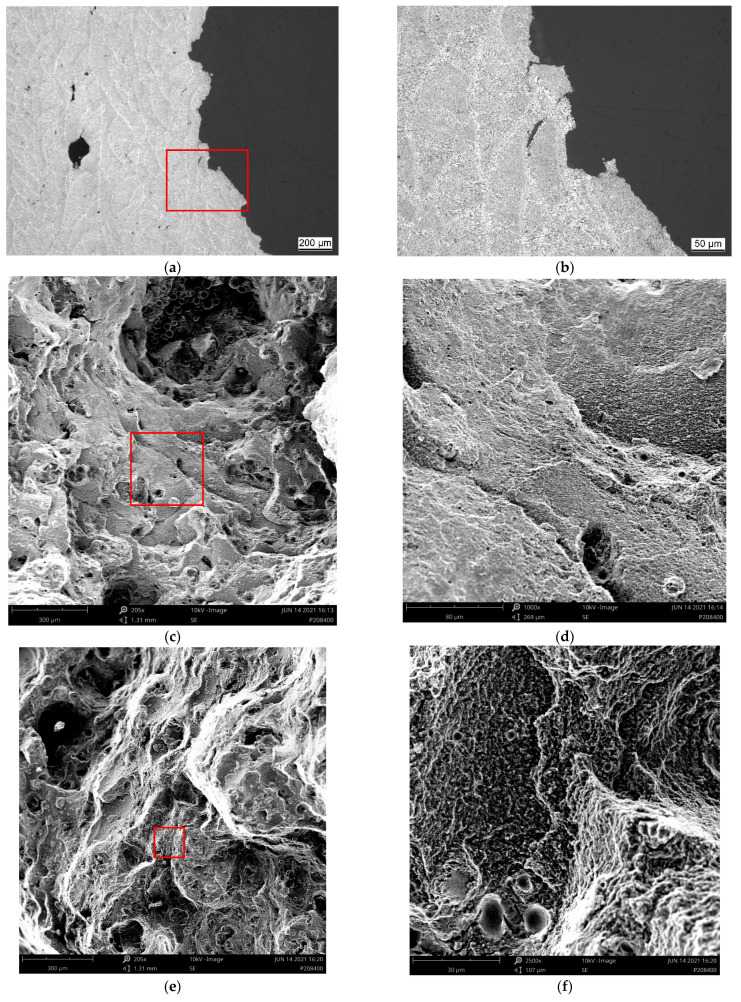
Region of fracture of a sample of polar angle 0° tested at room temperature: (**a**,**b**) metallographic section of etched samples at different magnifications, (**c**,**d**) back-scattered electron (BSE) images of the fracture surface at different magnifications and (**e**,**f**) secondary electron (SE) images of the fracture surface, overview and detailed view. Note that the red box in the image on the left highlights the area depicted in the magnified view on the right.

**Figure 9:**
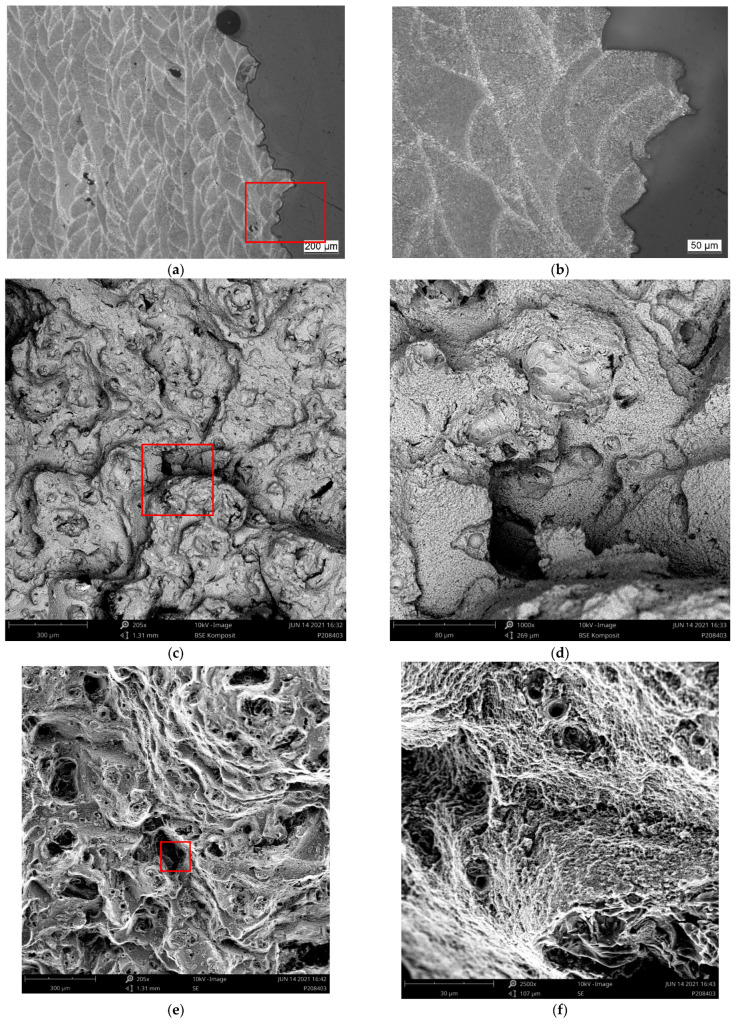
Region of fracture of a sample of polar angle 0° tested at 125 °C: (**a**,**b**) metallographic section of etched samples at different magnifications, (**c**,**d**) back-scattered electron (BSE) images of the fracture surface at different magnifications and (**e**,**f**) secondary electron (SE) images of the fracture surface, overview and detailed view. Note that the red box in the image on the left highlights the area depicted in the magnified view on the right.

**Figure 10:**
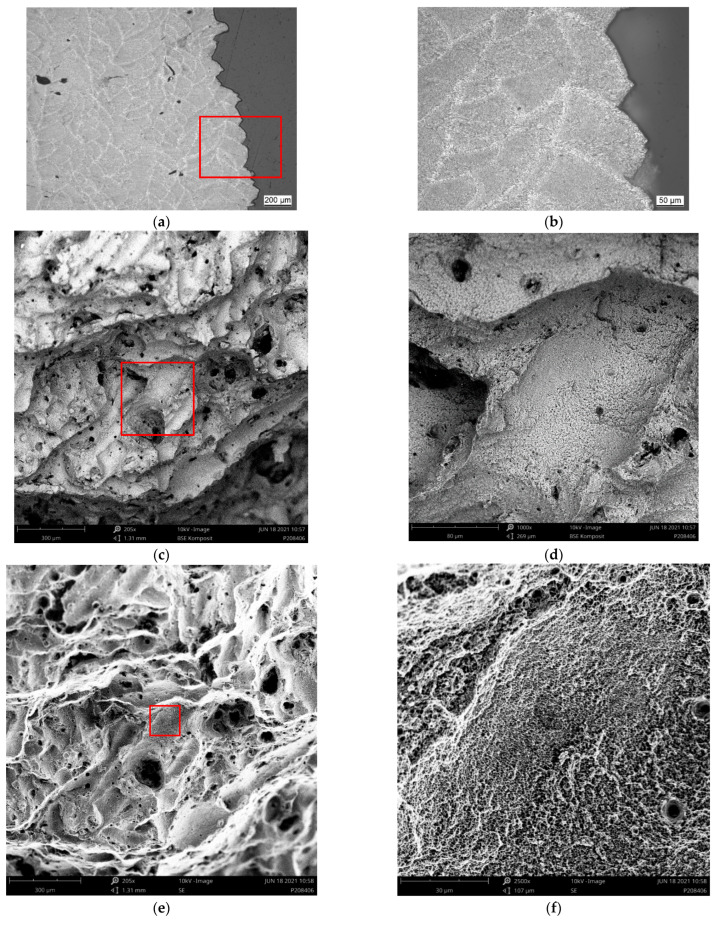
Region of fracture of a sample of polar angle 0° tested at 250 °C: (**a**,**b**) metallographic section of etched samples at different magnifications, (**c**,**d**) back-scattered electron (BSE) images of the fracture surface at different magnifications and (**e**,**f**) secondary electron (SE) images of the fracture surface, overview and detailed view. Note that the red box in the image on the left highlights the area depicted in the magnified view on the right.

**Figure 11:**
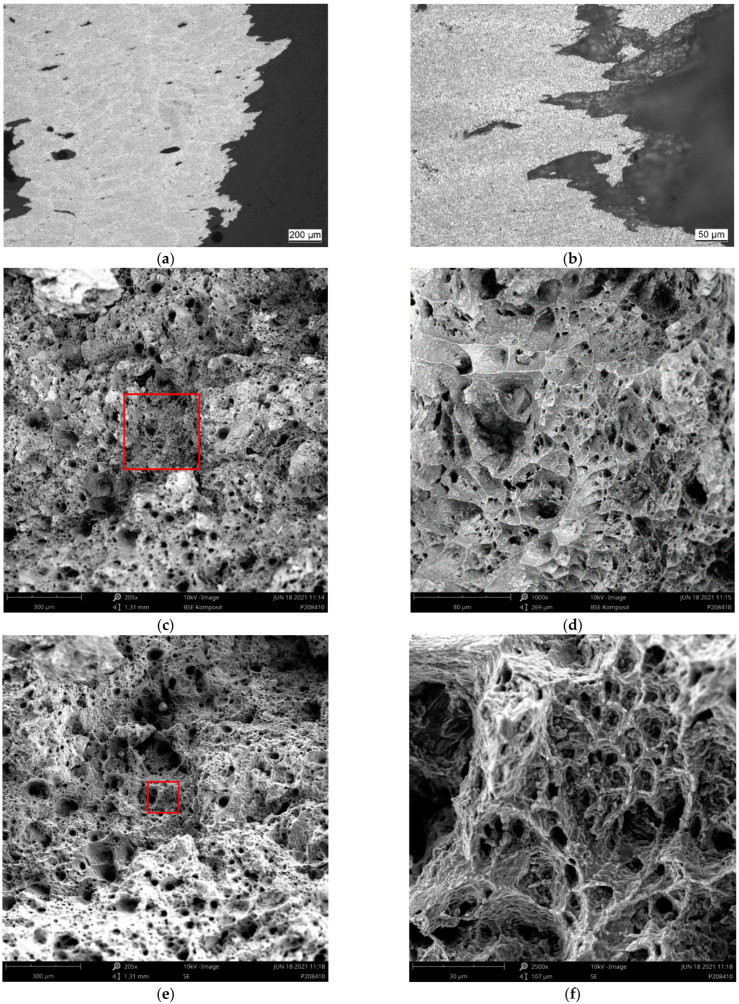
Region of fracture of a sample of polar angle 0° tested at 450 °C: (**a**,**b**) metallographic section of etched samples at different magnifications, (**c**,**d**) back-scattered electron (BSE) images of the fracture surface at different magnifications and (**e**,**f**) secondary electron (SE) images of the fracture surface, overview and detailed view. Note that the red box in the image on the left highlights the area depicted in the magnified view on the right.

Figure 8, Figure 9, Figure 10 and Figure 11 depict detailed views of the above metallographic sections focusing on the fracture surface, as well as SEM images of the fracture surface itself. The images are all organized in the same way: the left column is reserved for lower-, the right one for higher-magnification images. Wherever possible, a red square in the left column highlights the area corresponding to the detailed view seen on the right. The first row is reserved for metallographic sections, the second for fracture surface SEM images based on back-scattered electrons (BSE), while the final row displays similar secondary electron (SE) SEM images.

As in Figure 7, in Figure 8a,b, Figure 9a,b, Figure 10a,b and Figure 11a,b, the various tracks associated with the additive manufacturing process are clearly visible and delimited from each other by melt pool boundaries. Samples tested at room temperature and at 125 °C showed some indication of failure occurring along the melt pool boundaries. This is obvious in Figure 8b, where a sub-surface crack following such a boundary is clearly visible, as well as in Figure 9a,b, where the fracture surface roughly follows the network of the melt pool boundaries. However, the effect is most obvious in Figure 10a,b. Here, the line of failure as seen in the cross-section directly follows the aforementioned boundaries. This interpretation is supported by views of the fracture surfaces themselves. In Figure 10c,d, the imprint of the torn out melt tracks is clearly visible, which is not the case for samples tested at room temperature or at 125 °C. Room temperature samples (Figure 8) showed no indication of plastic deformation on the fracture surface, while some area fractions exhibiting ridges (Figure 9 and Figure 10, especially (e), (f)) suggest some such contribution to failure in the case of samples tested at intermediate temperatures.

The picture changes once again at the highest test temperature of 450 °C. Here, the failure is highly ductile, as is also reflected in the elongation at failure values measured at this temperature level, which exceed those associated with a test temperature of 250 °C by 69.4% for the respective polar angle (see Figure 4 as well as Table A2 for the actual values). Furthermore, the fracture surface as captured by the SEM images clearly shows the ductile dimples characteristic of this type of failure. These are lacking in all other cases with polar angle 0° irrespective of the test temperature, despite the fact that the sample drawn at 250 °C shows clear signs of plastic deformation in the form of necking (see Figure 6 and Figure 7). At 125 °C, the stress–strain curves depicted in Figure 4b suggest at least a slight effect of the same type, which is however not clearly expressed in the macroscopic depictions of metallographic sections (Figure 6 and Figure 7b). 

Porosity may be assumed to influence failure, specifically at the lower test temperatures, as pores were found on all fracture surfaces in these cases. In contrast, for specimens tested at 450 °C, Figure 11a,b reflect the elongation of such pores in the direction of the tensile load during plastic deformation. They do not, however, show clear evidence of fracture initiation as in the case of test conditions coinciding with less ductile material states (see Figure 8a). It may thus be assumed that the ductility of the material at the highest temperature incorporated in this study facilitates an alleviation of local stress concentrations around pores. In all other cases, the slightly higher porosity of the outer layer of the samples typically accounted for crack initiation.

**Figure 12:**
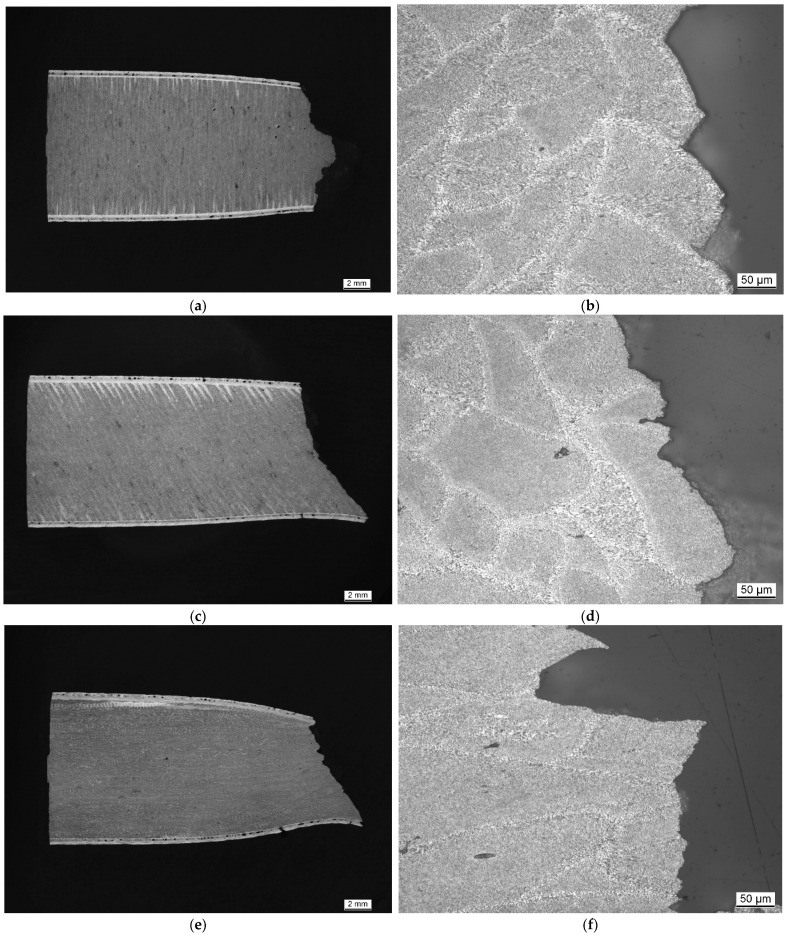
Metallographic sections of etched samples produced at varied polar angles after tensile testing at 250 °C. Left, general overview, right, detailed view of the fracture zone: (**a**,**b**) polar angle 0°, (**c**,**d**) polar angle 45 ° and (**e**,**f**) polar angle 90°.

Figure 12 contrasts samples of build orientations (polar angle) 0°, 45° and 90°, all tested at 250 °C. The macroscopic cross-sectional views suggest a predominantly brittle failure in the case of the 0° sample. Here, the fracture surface is roughly perpendicular to the direction of force, and though some necking is observable, its level is lower than in the case of the other two samples. The 45° polar angle indicates an origin of fracture from the surface and a transformation of global failure from a brittle mode to a ductile one, controlled by shear stresses and thus oriented at an angle of 45° relative to the direction of force. The amount of necking assumes an intermediate position between the 0° and the 90° polar angle samples. The latter exhibits a fracture surface with a clear 45° overall orientation. Failure is thus likely dominated by shear rather than normal stress, with the former being the largest in this inclined plane.

**Figure 13:**
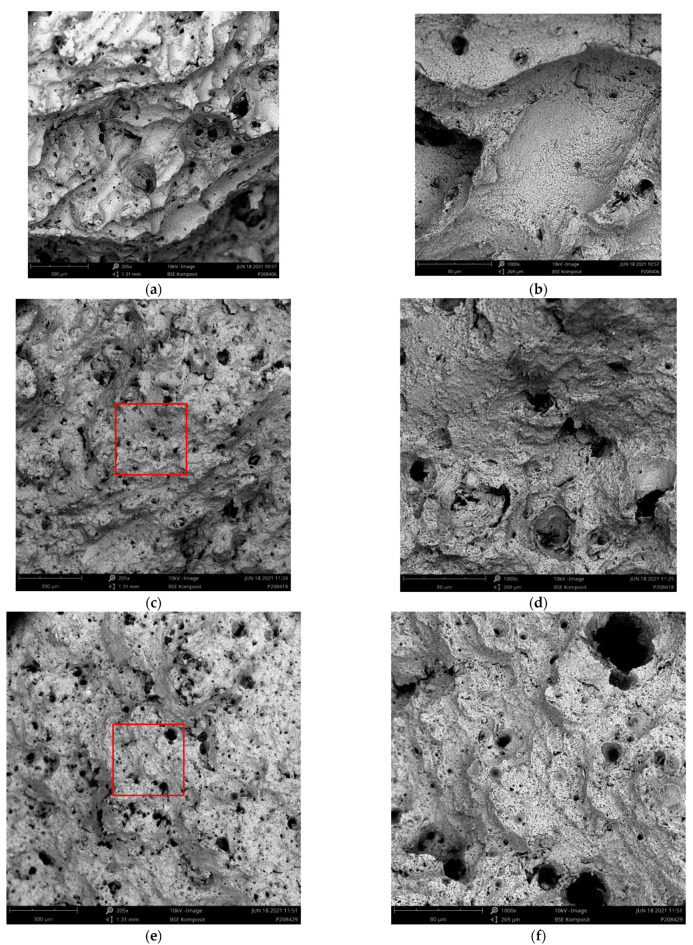
Fracture surfaces of samples produced at varied polar angles after tensile testing at 250 °C. Left column, low magnification overview, right, increased magnification: (**a**,**b**) polar angle 0°, (**c**,**d**) polar angle 45 ° and (**e**,**f**) polar angle 90°. Readers should note that the images in Figure 11a,b have already been shown as Figure 8c,d and are repeated here for direct comparison with their counterparts with polar angles 45° and 90°. Figures highlight the differences in failure locations relative to melt pool features also visible in the metallographic sections in Figure 12, specifically when comparing Figure 13a,b to Figure 13c–f. Note that the red box in the image on the left highlights the area depicted in the magnified view on the right.

Fracture surfaces as depicted in Figure 13 show two different appearances: At the 0° polar angle, individual tracks (melt pools) are visibly torn out, confirming the observation made on the metallographic sections in Figure 12 that in this case, fracture follows melt pool boundaries. In contrast, the fracture surfaces of samples printed at 45° and 90° polar angles resemble each other. In neither case could boundaries of individual tracks be distinguished on the fracture surface, which is irregular, but homogeneous on a macroscopic scale. All three fracture surfaces feature a significant number of pores, which appears to exceed the level of porosity seen in Figure 12. This is a possible indication of fracture following a weakest link, as defined by the spatial distribution of pores within the respective samples. 

**Figure 14:**
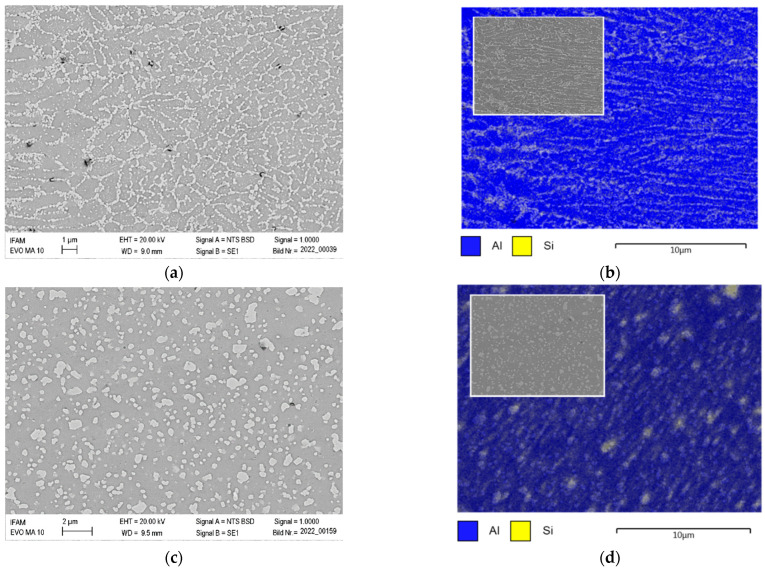
Microstructure of: (**a**,**b**) microstructure (**a**) and EDX area scan (**b**) for a sample produced at a 0° polar angle and tested at room temperature, and (**c**,**d**) microstructure (**c**) and EDX area scan (**d**) for a sample produced at a 0° polar angle and tested at 450 °C. Image is contrast-enhanced by 30% using the MS Word image formatting feature to increase material contrast in (**a**,**c**). Identification of bright-grey phases with silicon is confirmed. Images (**c**,**d**) confirm dissolution of the cellular structure as well as growth of the silicon phase.

Figure 14 depicts the microstructure of samples tested at room temperature (a) and at 450 °C (c). Of these, the former displays the transition from fine to coarse microstructure in the melt pool, while the latter shows a largely homogeneous distribution of separate phases (Figure 14a, right to left). The micrographs are accompanied by EDX area scans which highlight element distributions for aluminum and silicon measured on the same samples (b, d). Based on these, the light-gray phase in the former images can unequivocally be identified as silicon. The images suggest that the typical network-like or cellular arrangement of the Al-Si eutectic surrounding the α-Al phase, which is well-known from the literature [15,46,47,48], is still present in samples subjected to the stress-relieving treatment (2 h at 350 °C), however not retained in samples tested at temperatures of 450 °C. Hyer et al. have studied the expression of this network as a function of the primary process parameters scan speed and layer thickness, observing mesh sizes roughly between some tenths and 2–3 µm, and thus in good agreement with our own data, as seen in Figure 14a [15].

**Figure 15:**
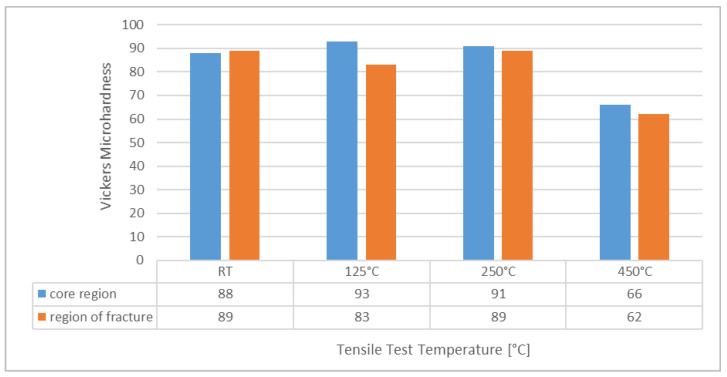
Microhardness measured near the fracture surface and in the sample core for specimens of polar angle 0° tested at room temperature (RT), 125, 250 and 450 °C. Note that the hardness measurements themselves were performed at room temperature.

Figure 15 contrasts the results of Vickers microhardness measurements performed on samples tested at various temperatures both in the sample core and directly adjacent to the fracture zone. The measurements show no obvious variation of hardness depending on the location within the sample, suggesting that in neither sample did significant strain hardening occur or was retained. Besides, the order of magnitude of the hardness data is similarly independent of the test temperature for RT, 125 and 250 °C. This finding matches the observation that for all these test temperatures, samples exhibit similar microstructures resulting from the stress-relief treatment, which is characterized by the typical eutectic network. The span of indentation sizes extended from 44.39 to 55.01 µm over all specimens and measurement positions and thus exceeds that of the cells by one order of magnitude. It is thus reasonable to conclude that the network of eutectic phases primarily determines the hardness values. Consequently, its dissolution leads to a significant reduction of this property: the sample tested at 450 °C exhibited a drop in hardness of approximately 40% compared to all other samples. The quantitative data thus confirm the microstructural observation (Figure 8a,b, Figure 9a,b, Figure 10a,b, Figure 11a,b and Figure 14) that up to temperatures of 250 °C, no significant change in material state is induced by the test conditions.

## 4. Discussion

In general, a review of the gathered room temperature mechanical properties shows good agreement with literature data for similar processing conditions. For thermal treatments of 2 h at temperatures between 275 and 300 °C, the overview in Appendix A relates UTS values between 247 and 345 MPa, yield strengths extending from 152.7 to 198 MPa and, in declining order, elongation at failure between 23.5% and 6.5% [14,21,22,32,36]. Note, though, that in all these cases, the build platform temperature was either given as ranging between RT and 80 °C, or not related at all, and further boundary conditions such as polar and azimuth angle or scanning parameters varied. In contrast, data for build platform temperatures of 200 °C, but with no further heat treatment, cover the regions of 314.32 to 349 MPa (UTS), 179.71 to 210 MPa (YS) and 3.2% to 5.9% (EaF) [16,31]. The corresponding values determined in the present study are 280.2 MPa (UTS), 165.94 MPa (YS) and 6.37% (EaF) at a polar angle of 0°.

Less sources report high-temperature properties, and even fewer do so for material conditions, i.e., heat treatment states, directly comparable to those studied in the present case. Liu et al. have tested as-built AlSi10Mg samples at 200 °C, determining YS, UTS and EaF at 193.83 MPa, 193.85 MPa and 17.2%, respectively, thus roughly matching our own data despite the fact that at room temperature, UTS levels determined by Liu et al. exceed our measurements by approximately 100 MPa [39]. Cao et al. studied as-built samples at 25, 100, 200, 300 and 400 °C, observing a decrease in UTS from 460 to 382, 266, 150 and 30 MPa and in YS from 322 to 298, 236, 143 and 25 MPa, accompanied by a rise of EaF from 6.94% to 13.14%, 23.73%, 26.56% and finally 75.02% [41]. Uzan et al. cover the same temperature range and find UTS dropping from 358 to 14 MPa and YS from 204 to 12 MPa, while EaF rises from 7.2% to 57.4% [40]. In all cases, it is noteworthy that despite the higher starting points (room temperature strength), property levels roughly meet with our own data at the highest temperatures. 

The original assumption that increased oxide levels caused by working with reused powders might positively affect high-temperature properties was not substantiated by the investigations. Results of the oxygen content measurements reported in Section 2 do not support an influence of this parameter on mechanical performance. At 0.042 wt.%, the levels undercut those reported by Raza et al. for virgin powder and are far below the levels nearing saturation detected on powders reused for 30 months, which reached in excess of 0.12%. Measurements are subject to some reservation, however, as the respective publication does not clearly state whether the data represent atom or wt.% [23]. However, in a further study, Weiss et al. reported oxygen contents of approximately 0.085 wt.% for virgin powder, increasing to roughly 0.092 wt.% after 10 reuse cycles, once more indicating that the material used in the present study can be assumed to match virgin powders in this respect. The study by Weiss et al. also considered Al, Si, Mg and hydrogen content, showing significant variation in the case of oxygen only. Within this somewhat higher range of oxygen content, Weiss et al. found no significant influence on density of LPBF samples and only a slight increase in Vickers hardness, with the latter exhibiting strong scatter [24].

**Figure 16:**
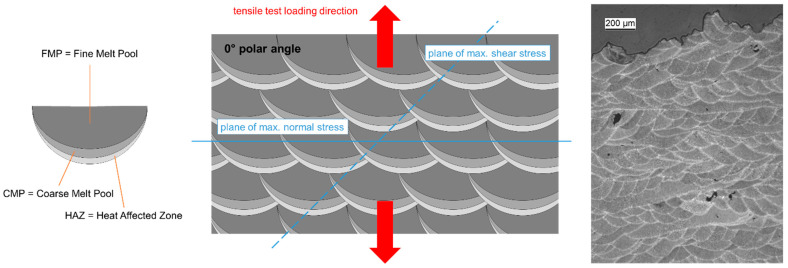
Microstructure of a sample printed at a 0° polar angle and cut in the *xz* plane assuming laser scanning in *y*-direction. The difference in comparison to the micrograph is due to the fact that for one thing the schematic representation does not account for the 60° layer-to-layer shift of the scanning direction, and besides the orientation of the micrograph is at an arbitrary angle to the scanning direction(s). The sketch to the left depicts the main microstructural zones based on the designation by Santos Macias et al. [43].

Figure 16 shows a simplified, schematic image of the typical microstructure of an AlSi10Mg sample produced via the LPBF process. In the adjacent micrograph, the well-known fish scale structure is less clear due to the layer-to-layer shift of the laser scanning trajectory by 60°. Santos Macias et al. trace the origins of the fine melt pool (FMP) zone to the melted powder layer plus 1–3 remelted layers below it, depending on process parameters, while the coarse melt pool (CMP) zone is considered a partially remelted section of the previously fused layer below the actual melt pool. The heat-affected zone (HAZ) follows further below and marks the region in which thermal energy input through the passing laser is still high enough to afford diffusion- and precipitation-based microstructural change, specifically in silicon phases both in the eutectic and precipitated from the aluminum solid solution. Both FMP and CMP zones are characterized by a 3D network or cellular structure of eutectic phase surrounding the aluminum grains [33,49,50,51]. Due to the high cooling rates typical of the LPBF process [52], the latter are not in equilibrium state, but represent a supersaturated solid solution [20]. The degree of supersaturation, i.e., the amount of elements solved, is controlled by the actual cooling rate and thus decreases with the increasing build platform temperature. Cell sizes also mainly depend on factors affecting the cooling rate and range roughly between 0.5 and a few µm [15,49].

When analyzing the elevated temperature property data gathered in the preceding section more closely, it must be considered that the heating-up to test temperatures and the dwell time required to guarantee thorough heating of the samples constitute a second heat treatment which adds to the effects of the initial stress relieving at 350 °C for 2 h. For obvious reasons, this effect is more relevant the higher the test temperature is. This is specifically true for testing at 450 °C, as this is the only temperature level that actually exceeds the stress-relief temperature. In fact, as Figure 14 shows, this secondary treatment has a significant effect on the structure of the eutectic phase, with its original network or cellular structure being dissolved to form larger, more globular silicon particles embedded in the Al matrix. The typical fish scale structure is visible in metallographic sections perpendicular to the scanning direction, which is schematically depicted in Figure 16, and thus the distinction between FMP, CMP and HAZ zones is less pronounced in these samples (see, e.g., Figure 14). For no other test temperature has such a severe transformation been observed. Similar overall effects have been reported by several authors for thermal treatments of additively manufactured AlSi10Mg samples at comparable temperatures of 200 °C and above.

Merino et al. did not observe structural change neither in melt pool patterns nor in eutectic cellular networks for heat treatments at 190 and 285 °C for slightly elevated build platform temperatures of 80 °C [22]. Pan et al. detected initial, though only very slight, signs of cellular network degradation already after 2 h of treatment at slightly lower temperatures of 275 °C. The fact that the cell size is approximately 500 nm suggests high cooling rates, as would be expected from a build platform kept at room temperature [36]. Zhang et al. compared the microstructures and mechanical properties of AlSi10Mg samples annealed for 2 h at 260, 280, 300 and 320 °C. Despite similar levels of laser power and scanning speed, in contrast to our own study, their investigations showed blurring of the typical fish-flake structure already at an annealing temperature of 260 °C. Coarsening of the eutectic and precipitation and growth of Si phases occurred in parallel. No mention is made of the build platform temperature, though [53]. Zhao et al. found size increases in Si precipitates already at heat treatment temperatures of 250 °C, and globularization as well as separation at 300 °C [54]. Deviation of these observations from our own may once more be related to the higher build platform temperature maintained in our study. Higher cooling rates reached via a room temperature build platform would naturally result in finer microstructures and higher levels of supersaturation in the aluminum phase, and thus an increased driving force for silicon redistribution, an effect confirmed in principle, e.g., by Santos Macias et al., who compared build platform temperatures of 35 and 200 °C [49]. Right from its start, growth of Si phases necessarily also results in a loss of connectivity within the cellular eutectic network, as has been quantitatively investigated by both Santos Macias et al. and Zhao et al. [49,54]. 

Fiocchi et al. followed a different path by performing thermal analyses of as-built AM-AlSi10Mg samples [20]. In differential scanning calorimetry (DSC) measurements, they identified two exothermal events which they can associate, via determination of the associated activation energies, with precipitation of Mg_2_Si phases and Si interdiffusion in Al, respectively. The latter has been proposed as the dominant mechanism behind Si particle growth by Ogris et al. in contrast to surface self-diffusion [55,56]. Extrapolation of rate-dependent DSC data allowed Fiocchi et al. to determine lower limit temperatures from which onwards these phenomena may be expected to occur. For Si diffusion, this margin is thus set at 294 °C, while the precipitation events already start at 263 °C. Experimental heat treatments at 263, 294 and 320 °C confirmed the theoretical conjecture, showing breakdown of the cellular network for the two higher-temperature treatments, accompanied in both cases by Si precipitation at the outer boundaries of the Al phase. This latter localization leads to traces of the original network structure still being visible in metallographic sections [20]. Fiocchi et al.’s results thus slightly deviate from our own, which advocate retention of the cellular network even after treatment at 350 °C (see Figure 14a,b) [20]. However, this apparent contradiction can be explained by the higher build platform temperature in the case of the present study, which resulted in a slightly coarsened cellular network and a lower degree of supersaturation of the Al phase. Both aspects will reduce the driving force for microstructural change.

Merino et al. as well as Rosenthal et al. thoroughly investigated fracture surfaces, detecting imprints of the cellular network on fracture surfaces resembling ductile dimples for samples heat-treated up to 285 °C in the case of Merino et al., who also determined cell and dimple sizes between 0.75 and 0.95 µm for annealing for 2 h at 190 and 285 °C, respectively [14,22,34]. These measurements are well-matched with our own data, which suggest cell and dimple sizes around 1 µm (see, e.g., Figure 10f and Figure 14a) for test temperatures below 450 °C.

These microstructural effects as such are not limited to additively manufactured Al-Si alloys containing eutectic phases but have been observed and studied in detail for cast materials of similar or identical composition, though often looking at typical solution heat treatment temperatures for this class of alloys above 500 °C [56,57,58]. Ogris et al., however, have discussed silicon spheroidization in Al-Si alloys in the temperature range of 400 to 540 °C for exposure times up to 12 h and proposed a model yielding the time to spheroidization of what they describe as the coral-like Si phase as a function of temperature. The validation experiments were performed on an Sr-modified A356 alloy, thus guaranteeing a comparatively fine expression of the eutectic Si, which, however, does not reach the size ranges typical of the eutectic in the fine melt pool zone of a similar, additively manufactured alloy. Nevertheless, even for such coarser Si structures, the disintegration time was found to be roughly 8 min at 450 °C, and still no more than approximately 19 min at 400 °C [56]. This explains the observed effect of testing at 450 °C on the microstructure, as depicted in Figure 14. Applying the model to the lower temperature level of the stress-relief treatment performed at 350 °C, spheroidization times approached 50 min, and thus fall short of the actual dwell time of 2 h. This explains why test conditions below 350 °C did not show any additional effect in terms of microstructural features or hardness, but also underlines that the initial stress-relieving must already have had an impact on sample microstructure—a fact also reflected in the comparatively low room temperature strength determined in the present study. 

It is thus obvious that the microstructural transformation must have an influence on mechanical properties. On the microscale, the dissolution of the original, three-dimensional network is likely to facilitate plastic deformation, while on the mesoscopic scale, the leveling-off of the distinctive features delimitating CMP, FMP and HAZ, which were still present in samples tested at up to 250 °C, could be expected to alleviate orientation dependence, as microstructural homogeneity is increased. In the latter respect, the differences in fracture surfaces between samples tested at RT, 125 and 250 °C compared to those subjected to testing at 450 °C is noteworthy: While the former show no or only very limited evidence of plasticity on the microscopic scale, the discontinuity of the embedded silicon phase in the latter as opposed to the cellular network of the eutectic phase allows for the ductile aluminum matrix to dominate deformation characteristics, as is evidenced by the strongly expressed ductile dimples seen, e.g., in Figure 11c–f. This notion is indirectly confirmed by several authors, who suggest that the highly interconnected, cellular structure formed by the eutectic in FMP and CMP regions acts as a failure initiation site when loaded in tension. Zhao et al. discussed this in detail, providing ample microscopic evidence, also including microstructures featuring isolated, globularized Si phases. At high connectivity of the eutectic phase, in this case in samples tested as-built and after 2 h of heat treatment at 250 °C, they found Si network breakage within the eutectic cell walls to be the dominant failure mechanism, with occasional nano-cracks transgressing the aluminum grains, though cracks mostly propagate via the eutectic. In contrast, in materials annealed for 2 h at 300 °C, which in this case show low-to-no Si connectivity and isolated Si particles, loss of cohesion between these and the Al matrix and eventual coalescence of such pores to form cracks transgressing the Al phase controls damage [54]. With the increasing size of the Si particles, fracture of these phases comes in as a further mechanism. Zhao et al. also identified typical strain levels for the respective processes and associated the lowest to the phenomena linked to the cellular network [50]. Aboulkhair et al. supported these findings by showing that as-built samples fracture along melt pool boundaries, while samples subjected to a T6 heat treatment (1 h at 520 °C, 6 h at 160 °C) fail via initiation of micro-voids at Si particles and merging of these [59,60]. Similarly, Zhao et al. found an accumulation of damage in the CMP area in samples with a predominantly intact fish scale and cellular eutectic structure [54]. These results align well with the fracture surface appearance of samples tested at 450 °C (see Figure 11), which is characterized by ductile dimples of roughly 5 to 15 µm in size, likely originating from individual, either fractured or de-bonded Si particles.

Besides the temperature influence, fracture surfaces of samples built at different orientations showed major deviations in terms of failure locations. The fact that samples produced at a polar angle of 0° clearly failed along melt pool boundaries when tested at 250 °C matches the findings of other authors [14,19,34]. What is striking, though, is that failure locations differed when comparing (a) different build orientations at an identical test temperature (see Figure 12 and Figure 13) and (b) different test temperatures (see Figure 8, Figure 9, Figure 10 and Figure 11). It must thus be assumed that the temperature dependence of the strength of the predominant microstructural zones differs in such a way that at 250 °C and under predominantly perpendicular load, CMP and/or HAZ regions are more prone to fail than the bulk FMP structure, whereas this order tends to be reversed at all other temperatures. 

A qualitative explanation may be derived from Figure 16, which illustrates that for samples built at polar angles of 0°, major portions of FMP, CMP and HAZ zones are effectively stacked and thus experience the same stress level. In contrast, at polar angles of 90°, mostly parallel orientation of these regions results in matching strain. For the intermediate polar angle of 45°, the orientation of these assumed layers coincides with the maximum shear stress plane. Thus, at a 0° polar angle, the combination of low ductility induced by the cellular network and lower strength linked to its coarse structure when compared to the FMP zone would make the CMP zone the logical failure location and thus explain the imprints of the melt pools on the fracture surfaces, e.g., as seen in Figure 10c,d. The fact that this phenomenon was most clearly expressed in the sample tested at 250 °C is assumed to be based on the differential softening of the HAZ as opposed to the CMP and FMP zones, which are dominated by the eutectic network rather than the Al matrix. However, this conjecture would suggest an increase of orientation-related differences in strength with increasing temperature, which cannot clearly be confirmed by the data gathered. Similarly, the tensile test results (UTS and YS values) do not confirm a generally higher orientation dependence at low test temperatures compared to 450 °C, a temperature for which microstructural observations show greater homogeneity.

## 5. Conclusions

The present study adds to the very limited number of studies on high-temperature characteristics of additively manufactured AlSi10 Mg by included previously unavailable information on the respective properties of stress-relieved materials. This is of specific interest for any application targeting elevated-temperature usage of such materials, as this could otherwise induce distortion through the release of residual stresses. Not surprisingly, the evolution of strength levels with temperature showed a constant decrease, with a considerable drop occurring between 250 and 450 °C data, leading to marginal levels between 17.52 and 39.30 MPa, depending on orientation, at the latter temperature. The respective step, when averaging data over all orientations, amounted to a decrease by 81.7% and thus exceeded that observed for the similar temperature range between room temperature and 250 °C, which reached 38.8%, by a considerable margin (averaged UTS at RT 271.5 MPa, 166.1 MPa at 250 °C and 30.4 MPa at 450 °C, with the respective YS values being 155.4, 134.5 and 29.0 MPa). This observation can be explained based on microstructural change, which suggested a strong driving force for Si modification at and above 350 °C—the stress-relieving temperature applied—which was confirmed by the relevant literature. For further studies, it might thus be interesting to investigate the critical temperature range more closely by adding further data points in the interval between 250 and 450 °C. The effect as such also highlights a difficulty of the present approach, as it must be assumed, and can be substantiated by microscopic evidence, that the tensile test conditions at 450 °C, and here both the soaking and the actual testing time, will affect microstructural characteristics. This implies that the material condition tested does not exactly match the initial one, and thus also not that of the samples tested at lower temperatures.

From the application perspective originally motivating this research, i.e., compound casting for integration of cooling channels in cast metal components and the ensuing need for high-temperature strength, it would be interesting to clarify which duration of high-temperature immersion suffices to alter the structure of the silicon phase and thus affect strength levels, as observed in the present study. Due to the high cooling rates associated with the HPDC process envisaged for production of such components, real-world conditions will exceed the temperatures covered in the present investigation, but fall short of the times reached during tensile testing at 450 °C as reported here. Some background information on the temperature dependence of Si coarsening has been gathered by Ogris et al., but the experimental data backing the theoretical model do not necessarily warrant extrapolation to short exposure times, justifying further scrutiny [56].

In terms of the property levels observed, the general observation (see Figure 1) that material conditions after stress relieving do not excel in terms of strength has been confirmed. As these processing conditions were chosen to reduce residual stress levels, a recent publication by Van Cauwenbergh et al. is of interest, claiming that warm aging of as-built AlSi10Mg for 6 h at 170 °C to achieve a T5 state will reduce residual stresses just as much as stress relieving at much higher temperatures of 270 or 300 °C for 2 h does [32]. The advantage of Van Cauwenbergh et al.’s procedure would be that the T5 treatment allows to retain or even slightly increase the mechanical properties of as-built samples, as has been shown by Van Cauwenbergh et al. themselves as well as several other authors [14,32,34,35,37]. The big questions related to this point are: (a) whether this superior strength can be maintained at all under conditions of high-pressure die casting, and (b) if this is not the case, if the higher strength of the material in the temperature ramp-up phase during casting might still have beneficial effects in terms of pushing the sustainable pressure of HPDC inserts towards higher limits.

Future studies should thus both shed more light on the kinetics of microstructural change and its effect on high-temperature properties, and include T5 or warm-aged samples to quantify their performance under elevated-temperature conditions, while new alloy compositions tuned towards improved high-temperature strength could be added. The respective findings would support optimized layout of additively manufactured cooling channels and other components for integration in HPDC parts. Combining these two processes via compound casting would then finally open up new application perspectives for AM, bringing together the geometrical flexibility of the LPBF process with the productivity of high-pressure die casting, thus facilitating highly performant, but nevertheless economically viable components for the automotive and other industries.

## Figures and Tables

**Table 1:** Chemical composition of the AlSi10Mg powders and samples according to specification [43], supplier measurements and as determined on printed samples by OES.

Element	Spec. ^1^	Supplier Data ^2^	Measurement Results (wt.%)					
	(wt.%)	(wt.%)	#1	#2	#3	#4	av. ^3^	SD ^4^ (%)
Al	Balance	89.54	88.95	88.85	89.02	88.78	88.9	0.119
Si	9.0–11.0	9.70	10.605	10.69	10.561	10.786	10.661	0.932
Fe	<0.55	0.11	0.12	0.122	0.113	0.117	0.118	3.32
Cu	<0.05	0.05	0.0028	0.003	0.0021	0.0027	0.0027	14.62
Mn	<0.45	0.01	0.003	0.003	0.003	0.003	0.003	0
Mg	0.20–0.45	0.39	0.2773	0.2786	0.2709	0.2806	0.2769	1.514
Zn	<0.10	0.01	0.003	0.003	0.002	0.003	0.0028	18.18
Ti	<0.15	0.01	0.028	0.031	0.017	0.019	0.0238	28.63
Ni	<0.05	0.01	0.003	0.01	0.005	0.005	0.0058	51.93
Pb	<0.05	0.01	0	0	0	0	0	n.a.
Sn	<0.05	0.01	0	0	0	0	0	n.a.
Others	<0.15	0.15	0.0131	0.0156	0.0114	0.0125	0.0132	13.53

^1^ Powder supplier specification [43]. ^2^ Supplier measurement on actual powder batch. ^3^ Average of measurements. ^4^ Standard deviation, given as percentage of the average value.

**Table 2:** LPBF process parameters as used for sample contour and bulk in the present study.

Parameter (Unit)	Contour	Bulk
Laser Power (W)	370	370
Scan Speed (mm/s)	300	2000
Hatch Distance (µm)	- ^1^	90
Layer Thickness (µm)	60	60

^1^ Only a single pre-contour was produced, so hatch distance does not apply here.

**Table 3:** Comparison between properties of as-manufactured and turned samples in terms of yield strength, ultimate tensile strength and elongation at failure as measured at room temperature on samples with polar angle 0°. Note that a sample stress–strain curve for a turned sample is also included in Figure 4a.

Parameter (Unit)	As-Manufactured Samples	Turned Samples
Yield Strength, YS (MPa)	165.94	169.45
YS SD (MPa)	11.40	1.16
Ultimate Tensile Strength, UTS (MPa)	280.20	289.02
UTS SD (MPa)	0.69	0.93
Elongation at Failure (%)	6.37	8.40
EaF ^1^ SD (%)	0.19	0.91

^1^ Elongation at Failure.

## Data Availability

Data is published in the Appendix A. Any further information can be obtained from the authors on request.

## Appendix A

The table below lists the quantitative data used for the graphical representation of yield strength, ultimate tensile strength and elongation at failure of additively manufactured AlSi10Mg in Figure 1. Besides providing the actual data, additional information is presented regarding the main influencing factors, i.e., material conditions based on, e.g., heat treatments or other parameters affecting mechanical performance, such as processing conditions or build orientation. Furthermore, the related references are listed. Readers should note, though, that it is not possible to include all details of process parameters and hatching methods. For such details, we refer the reader to the original publications.

**Table A1:** Overview of ultimate tensile strength (UTS), yield strength (YS) and elongation at failure data as depicted graphically in Figure 1 with additional background information on material state, process parameters and boundary conditions. As definitions of polar and azimuth angles sometimes vary in the literature, these have been transformed to match the definitions provided in Figure 2 above. The data are sorted not by source, but in ascending order based on the reported UTS value.

UTS (MPa) (SD (MPa))	YS (MPa) (SD (MPa))	Elongation ^1^ (%) (SD (%))	Ref.	Comments, Processing Conditions (BP = Build Platform, PA = Polar Angle, AA = Azimuth Angle, SR = Stress-Relieved, SHT = Solution Heat-Treated, WA = Warm-Aged)
184 (1)	115 (5)	20.2 (0.4)	[34]	BP @ 35 °C, PA 0°, HIP ^2^ 2 h @ 500 °C, EOS M280, Ar atm.
241	154	24.1	[22]	BP @ 80 °C, PA 90°, P_Laser_ 300 W, v_S_ = 1 m/s, SR 2 h @ 285 °C, WA 1000 h @ 177 °C, EOS M290.
245	139	18.1	[21]	BP @ RT, PA 0°, SHT 6 h @ 530 °C, EOS M280, Ar atm.
245	155	14.8	[22]	BP @ 80 °C, PA 0°, P_Laser_ 300 W, v_S_ = 1 m/s, SR 2 h @ 285 °C, WA 1000 h @ 177 °C, EOS M290.
246	154	21.6	[22]	BP @ 80 °C, PA 0°, P_Laser_ 300 W, v_S_ = 1 m/s, SR 2 h @ 285 °C, WA 10 h @ 177 °C, EOS M290.
247	157	23.5	[22]	BP @ 80 °C, PA 90°, P_Laser_ 300 W, v_S_ = 1 m/s, SR 2 h @ 285 °C, EOS M290.
249	153	21.3	[22]	BP @ 80 °C, PA 0°, P_Laser_ 300 W, v_S_ = 1 m/s, SR 2 h @ 285 °C, EOS M290.
248	126	6.3	[26]	BP @ 220 °C, PA 90°, P_Laser_ 960 W, v_S_ > 1 m/s, tested as-built.
250	164	23.4	[22]	BP @ 80 °C, PA 90°, P_Laser_ 300 W, v_S_ = 1 m/s, SR 2 h @ 285 °C, WA 10 h @ 177 °C, EOS M290.
267.7	152.7	16.18	[32]	PA 0°, SR 2 h @ 300 °C, 3D Systems ProX^®^ DMP 320.
269	153	18.3	[21]	BP @ RT, PA 90°, SHT 6 h @ 530 °C, EOS M280, Ar atm.
271	174	16.5	[22]	BP @ 80 °C, PA 0°, P_Laser_ 300 W, v_S_ = 1 m/s, SR 2 h @ 285 °C, WA 100 h @ 177 °C, EOS M290.
273	180	20.9	[22]	BP @ 80 °C, PA 90°, P_Laser_ 300 W, v_S_ = 1 m/s, SR 2 h @ 285 °C, WA 100 h @ 177 °C, EOS M290.
285	180	18.6	[21]	BP @ RT, PA 90°, SHT 2 h @ 300 °C, EOS M280, Ar atm.
286 (2)	183 (5)	7 (0.2)	[34]	BP @ 35 °C, PA 0°, WA 2 h @ 300 °C, EOS M280, Ar atm.
290	175	14.2	[21]	BP @ RT, PA 0°, SHT 2 h @ 300 °C, EOS M280, Ar atm.
302	150	4.0	[26]	BP @ 220 °C, PA 0°, P_Laser_ 960 W, v_S_ > 1 m/s, tested as-built.
302 (1.4)	223 (2.8)	16.0 (2.5)	[35]	BP @ RT, PA 0°, SHT 1 h @ 540 °C, WA 4 h @ 160 °C.
302.4	174.1	11.68	[32]	PA 0°, SR 2 h @ 270 °C, 3D Systems ProX^®^ DMP 320.
308	221	11.8	[28]	BP @ 150 °C, PA 0°, SHT 1 h @ 540 °C, WA 4 h @ 160 °C, SLM500, Ar atm.
310 (3)	-	9.2	[14]	PA 90°, AA 0°, WA 2 h @ 300 °C, Concept Laser^©^ M2, Ar atm.
310.8 (1.3)	198.0 (2.0)	13.7 (0.6)	[36]	P_Laser_ 370 W, v_S_ = 1.454 m/s, SR 2 h @ 275 °C, Eplus3D EP-M250, Ar atm.
314.32 (7.24)	179.71 (8.31)	3.97 (0.45)	[16]	BP @ 200 °C, PA 45°, AA 5°, tested as-built, SLM 280HL.
319	251	12.6	[28]	BP @ 150 °C, PA 0°, SHT 10 min. @ 510 °C, WA 6 h @ 160 °C, SLM500, Ar atm.
323 (0.0)	243 (0.0)	15.3 (2.4)	[35]	BP @ RT, PA 90°, SHT 1 h @ 540 °C, WA 4 h @ 160 °C.
330.11 (10.39)	188.15 (7.04)	4.47 (0.15)	[16]	BP @ 200 °C, PA 45°, AA 0°, tested as-built, SLM 280HL
342	266	-	[18]	PA 90°, tested as-built, Renishaw AM400.
342 (5)	-	6.5	[14]	PA 0°, WA 2h @ 300 °C, Concept Laser© M2, Ar atm.
344	192	5.9	[31]	BP @ 200 °C, PA 45°, AA 90°, SLM 280HL.
344.73 (20.56)	198.13 (13.64)	3.2 (0.19)	[16]	BP @ 200 °C, PA 0°, AA 45°, tested as-built, SLM 280HL.
348	209	5.9	[31]	BP @ 200 °C, PA 90°, AA 0°, SLM 280HL.
349	210	4.4	[31]	BP @ 200 °C, PA 60°, AA 90°, SLM 280HL.
355 (5)	234 (5)	11 (2.2)	[22]	BP @ 80 °C, PA 90°, P_Laser_ 300 W, v_S_ = 1 m/s, SR 2 h @ 190 °C, WA 1000 h @ 177 °C, EOS M290.
356	250	-	[18]	PA 0°, tested as-built, Renishaw AM400.
357	254	-	[18]	PA 30°, tested as-built, SLM 280.
357.49 (19.60)	208.57 (16.94)	3.15 (0.08)	[16]	BP @ 200 °C, PA 0°, AA 5°, tested as-built, SLM 280HL.
360	239	-	[18]	PA 90°, tested as-built, SLM 280.
360.27 (10.44)	222.83 (9.30)	5.33 (0.46)	[16]	BP @ 200 °C, PA 90°, AA 85°, tested as-built, SLM 280HL
362	270	-	[18]	PA 30°, tested as-built, Renishaw AM400.
364	225	6.5	[31]	BP @ 200 °C, PA 90°, AA 90°, SLM 280HL.
366 (5)	245 (5)	10.8 (1.2)	[22]	BP @ 80 °C, PA 90°, P_Laser_ 300 W, v_S_ = 1 m/s, SR 2 h @ 190 °C, WA 10 h @ 177 °C, EOS M290.
366.43 (12.51)	206.74 (4.42)	6.12 (1.10)	[16]	BP @ 200 °C, PA 90°, AA 5°, tested as-built, SLM 280HL.
370	280	-	[18]	PA 90°, tested as-built, EOS M400.
372 (4)	248 (4)	10.4 (1.1)	[22]	BP @ 80 °C, PA 90°, P_Laser_ 300 W, v_S_ = 1 m/s, WA 1000 h @ 177 °C, EOS M290.
374	256	-	[18]	PA 30°, tested as-built, EOS M400.
376 (9)	250 (6)	10.1 (1.1)	[22]	BP @ 80 °C, PA 90°, P_Laser_ 300 W, v_S_ = 1 m/s, SR 2 h @ 190 °C, EOS M290.
377	205	3.3	[31]	BP @ 200 °C, PA 0°, AA 90°, SLM 280HL.
377 (11)	250 (5)	10.2 (1.1)	[22]	BP @ 80 °C, PA 90°, P_Laser_ 300 W, v_S_ = 1 m/s, SR 2 h @ 190 °C, WA 100 h @ 177 °C, EOS M290.
379	244	-	[18]	PA 0°, tested as-built, EOS M400.
379.67	226.63	8.18	[39]	PA not specified, P_Laser_ 200 W, Concept Laser M2, N_2_ atm.
383.67	247.67	9.833	[30]	PA 90°, P_Laser_ 370 W, v_S_ = 1.3 m/s, tested as-built, EOS M280.
386 (2.6)	248 (1.7)	8.6 (1.4)	[35]	BP @ 160 °C, PA 90°, tested as-built.
386	249	-	[18]	PA 0°, tested as-built, SLM 280.
387 (4)	221 (6)	5.3 (1.1)	[22]	BP @ 80 °C, PA 0°, P_Laser_ 300 W, v_S_ = 1 m/s, SR 2 h @ 190 °C, WA 1000 h @ 177 °C, EOS M290.
388 (7)	256 (8)	10.2 (0.9)	[22]	BP @ 80 °C, PA 90°, P_Laser_ 300 W, v_S_ = 1 m/s, WA 100 h @ 177 °C, EOS M290.
389 (4)	263 (3)	9.4 (0.8)	[22]	BP @ 80 °C, PA 90°, P_Laser_ 300 W, v_S_ = 1 m/s, WA 10 h @ 177 °C, EOS M290.
391 (6 ^3^)	-	5.55 (0.4 ^3^)	[29]	PA 90°, AA 0°, tested as-built, Concept Laser M1.
391 (5)	231 (9)	4.6 (0.7)	[22]	BP @ 80 °C, PA 0°, WA 1000 h @ 177 °C, EOS M290.
396 (8 ^3^)	-	3.47 (0.6 ^3^)	[29]	PA 0°, tested as-built, Concept Laser M1.
399.1 7.330	241.15 5.697	6.47 0.361	[16]	BP @ 200 °C, PA 90°, AA 5°, tested as-built, SLM 280HL.
401	214	3.2	[26]	BP @ RT, PA 0°, P_Laser_ 240 W, v_S_ = 0.5 m/s, tested as-built.
403 (9)	229 (12)	4.2 (0.6)	[22]	BP @ 80 °C, PA 0°, P_Laser_ 300 W, v_S_ = 1 m/s, WA 100 h @ 177 °C, EOS M290.
407 (8)	229 (7)	5 (0.7)	[22]	BP @ 80 °C, PA 0°, P_Laser_ 300 W, v_S_ = 1 m/s, SR 2 h @ 190 °C, WA 100 h @ 177 °C, EOS M290.
411 (9)	231 (7)	5 (0.8)	[22]	BP @ 80 °C, PA 0°, P_Laser_ 300 W, v_S_ = 1 m/s, SR 2 h @ 190 °C, WA 10 h @ 177 °C, EOS M290.
412 (5.5)	228 (4.1)	7.0 (0.1)	[35]	BP @ 160 °C, PA 0°, tested as-built.
415	231	10.5	[26]	BP @ RT, PA 90°, P_Laser_ 960 W, v_S_ > 1 m/s, tested as-built.
416	242	5.9	[26]	BP @ RT, PA 90°, P_Laser_ 240 W, v_S_ = 0.5 m/s, tested as-built.
418 (9)	233 (12)	5.1 (0.8)	[22]	BP @ 80 °C, PA 0°, P_Laser_ 300 W, v_S_ = 1 m/s, WA 10 h @ 177 °C, EOS M290.
421.67	239	5.176	[30]	PA 0°, P_Laser_ 370 W, v_S_ = 1.3 m/s, tested as-built, EOS M280.
436	203	5.5	[26]	BP @ RT, PA 0°, P_Laser_ 960 W, v_S_ > 1 m/s, tested as-built.
436.8	231.6	7.04	[32]	PA 0°, tested as-built, 3D Systems ProX^®^ DMP 320.
436.8	264.3	4.19	[32]	PA 0°, WA 6 h @ 170 °C, 3D Systems ProX^®^ DMP 320.
443 (16)	258 (4)	4.7 (1.2)	[22]	BP @ 80 °C, PA 0°, P_Laser_ 300 W, v_S_ = 1 m/s, SR 2 h @ 190 °C, EOS M290.
444.85 (8.73)	341.49 (9.97)	2.55 (0.27)	[33]	P_Laser_ 220 W, v_S_ = 1.1 m/s, tested as-built, DiMetal-100, Ar atm.
447	250	4.1	[28]	BP @ 150 °C, PA 0°, tested as-built, SLM500, Ar atm.
452	256	4.3	[28]	BP @ 150 °C, PA 0°, WA 4 h @ 160 °C, SLM500, Ar atm.
455 (12)	300 (3)	5.2 (0.6)	[27]	P_Laser_ 350 W, v_S_ = 1.17 m/s, SLM 250 HL.
457 (2)	274 (10)	12.4 (1.3)	[22]	BP @ 80 °C, PA 90°, P_Laser_ 300 W, v_S_ = 1 m/s, EOS M290.
459 (3)	-	6.8	[14]	PA 90°, AA 0°, WA 2 h @ 200 °C, Concept Laser© M2, Ar atm.
459.21 (13.77)	346.18 (10.45)	2.88 (0.29)	[33]	P_Laser_ 220 W, v_S_ = 1.1 m/s, tested as-built, DiMetal-100, N_2_ atm.
463 (3)	237 (4)	7.6 (1.0)	[22]	BP @ 80 °C, PA 0°, P_Laser_ 300 W, v_S_ = 1 m/s, EOS M290.
464 (2)	-	7.1	[14]	PA 90°, AA 0°, tested as-built, Concept Laser© M2, Ar atm.
468 (1)	-	5.4	[14]	PA 0°, tested as-built, Concept Laser© M2, Ar atm.
471 (0.8)	321 (1.8)	8.6 (0.5)	[35]	BP @ RT, PA 90°, WA 4 h @ 160 °C.
473 (1)	263 (5)	7.7 (0.1)	[34]	BP @ 35 °C, PA 0°, tested as-built, EOS M280, Ar atm.
475	279	7.5	[21]	BP @ RT, PA 90°, EOS M280, Ar atm.
476	220	5.5	[21]	BP @ RT, PA 0°, EOS M280, Ar atm.
478 (1)	-	3.0	[14]	PA 0°, WA 2 h @ 200 °C, Concept Laser© M2, Ar atm.
479 (2)	298 (5)	5.6 (0.1)	[34]	BP @ 35 °C, PA 0°, WA 2 h @ 200 °C, EOS M280, Ar atm.
483.3 (3.1)	289.3 (2.1)	7.2 (1.0)	[36]	P_Laser_ 370 W, v_S_ = 1.293 m/s, tested as-built, Eplus3D EP-M250, Ar atm.
489.45 (3.20)	323.75 (2.28)	4.51 (0.22)	[33]	P_Laser_ 220 W, v_S_ = 1.1 m/s, remelting step w. P_Laser_ 200 W, v_S_ = 1.2 m/s, tested as-built, DiMetal-100, Ar atm.
492	292	5.6	[38]	BP @ 150 °C, tested as-built.
493 (0.6)	292 (0.6)	6.0 (0.6)	[35]	BP @ RT, PA 0°, WA 4 h @ 160 °C.
493	296	5.0	[37]	BP @ 50 °C, WA 6 h @ 160 °C.
494.3 (1.2)	294.7 (2.5)	7.0 (1.3)	[36]	P_Laser_ 370 W, v_S_ = 1.344 m/s, tested as-built, Eplus3D EP-M250, Ar atm.
496.0 (2.6)	297.0 (1.0)	6.3 (0.3)	[36]	P_Laser_ 370 W, v_S_ = 1.511 m/s, tested as-built, Eplus3D EP-M250, Ar atm.
500.14 (5.15)	324.91 (4.82)	5.13 (0.27)	[33]	P_Laser_ 220 W, v_S_ = 1.1 m/s, remelting step w. P_Laser_ 200 W, v_S_ = 1.2 m/s, tested as-built, DiMetal-100, N_2_ atm.
500.7 (0.8)	311.5 (5.9)	7.7 (0.5)	[36]	P_Laser_ 370 W, v_S_ = 1.454 m/s, tested as-built, Eplus3D EP-M250, Ar atm.

^1^ Elongation at failure. ^2^ Hot isostatic pressing (HIP). ^3^ 95% confidence interval.

**Table A2:** Overview of the data measured in the course of the present study, grouped according to the test temperature.

Temperature (°C)	Polar Angle (°)	UTS (MPa) *SD* (MPa)	YS (MPa) *SD* (MPa)	Elongation ^1^ (%) *SD* (%)	E (GPa) *SD* (GPa)	Comments
RT	0	289.02 *0.931*	169.25 *1.16*	8.40 0.908	72.00 *1.18*	3 samples, surface turned.
	0	280.20 *0.691*	165.94 *11.40*	6.37 *0.193*	71.86 *1.32*	3 samples, surface as-built.
	45	272,02 *1.85*	154,94 *1.74*	8.06 *0.573*	78.40 *4.59*	3 samples, surface as-built.
	90	265.92 *9.43*	145.24 *11.15*	9.88 *4.65*	69.63 ^2^ *1.07*	5 samples, surface as-built.
125	0	249.68 *3.00*	154.98 *2.20*	7.66 *1.02*	70.41 *3.14*	3 samples, surface as-built.
	45	238.26 *9.23*	151.70 *4.11*	9.73 *1.15*	67.48 *7.66*	3 samples, surface as-built.
	90	239.37 *3.94*	155.39 0.94	9.63 *1.27*	63.03 *1.62*	3 samples, surface as-built.
250	0	162.82 *9.08*	132.95 *3.33*	15.64 *5.05*	55.74 ^3^ *1.39*	5 samples, surface as-built.
	45	174.11 *2.42*	135.75 *3.26*	14.60 *3.07*	50.74 *1.20*	3 samples, surface as-built.
	90	163.56 *18.79*	135.72 *9.08*	17.61 ^2^ *0.62*	46.91 ^2^ *1.63*	3 samples, surface as-built.
450	0	34.42 *17.94*	32.51 *17.85*	26.49 *9.67*	- ^4^	2 samples, surface as-built.
	45	39.30 *18.19*	37.96 *18.21*	30.79 *21.91*	- ^4^	2 samples, surface as-built.
	90	17.52 *0.28*	16.40 *0.78*	45.63 *10.47*	- ^4^	2 samples, surface as-built.

^1^ Elongation at failure. ^2^ Data from two samples only. ^3^ Data from four samples only. ^4^ No clear determination of Young’s modulus possible.
